# RNA-Seq Analysis Reveals a Six-Gene SoxR Regulon in *Streptomyces coelicolor*

**DOI:** 10.1371/journal.pone.0106181

**Published:** 2014-08-27

**Authors:** Nawar Naseer, Joshua A. Shapiro, Monica Chander

**Affiliations:** Department of Biology, Bryn Mawr College, Bryn Mawr, Pennsylvania, United States of America; Niels Bohr Institute, Denmark

## Abstract

The redox-regulated transcription factor SoxR is conserved in diverse bacteria, but emerging studies suggest that this protein plays distinct physiological roles in different bacteria. SoxR regulates a global oxidative stress response (involving >100 genes) against exogenous redox-cycling drugs in *Escherichia coli* and related enterics. In the antibiotic producers *Streptomyces coelicolor* and *Pseudomonas aeruginosa,* however, SoxR regulates a smaller number of genes that encode membrane transporters and proteins with homology to antibiotic-tailoring enzymes. In both *S. coelicolor* and *P. aeruginosa*, SoxR-regulated genes are expressed in stationary phase during the production of endogenously-produced redox-active antibiotics. These observations suggest that SoxR evolved to sense endogenous secondary metabolites and activate machinery to process and transport them in antibiotic-producing bacteria. Previous bioinformatics analysis that searched the genome for SoxR-binding sites in putative promoters defined a five-gene SoxR regulon in *S. coelicolor* including an ABC transporter, two oxidoreductases, a monooxygenase and an epimerase/dehydratase. Since this *in silico* screen may have missed potential SoxR-targets, we conducted a whole genome transcriptome comparison of wild type *S. coelicolor* and a *soxR-*deficient mutant in stationary phase using RNA-Seq. Our analysis revealed a sixth SoxR-regulated gene in *S. coelicolor* that encodes a putative quinone oxidoreductase. Knowledge of the full complement of genes regulated by SoxR will facilitate studies to elucidate the function of this regulatory molecule in antibiotic producers.

## Introduction

The redox-regulated transcription factor SoxR is present in a diverse range of *Proteobacteria* and *Actinobacteria* and homologs are highly similar at the amino acid level [1]. SoxR homologs function as homodimers and have a conserved amino-terminal helix-turn-helix DNA binding domain, suggesting that these proteins bind to and regulate transcription from similar operator sequences. This has been confirmed in organisms where SoxR has been biochemically characterized [2]–[7]. SoxR homologs also share a conserved sequence (CysX2CysXCysX5Cys) in the carboxy-terminus that has been shown to be necessary for coordinating [2Fe-2S] centers in SoxR proteins from *Escherichia coli, Pseudomonas aeruginosa,* and *Streptomyces coelicolor* [5], [8], [9]. These [2Fe-2S] clusters are central to SoxR’s ability to detect changes in the cellular redox environment and regulate gene expression in response. SoxR was originally discovered in the enterobacterium *E. coli* where it promotes resistance to redox-cycling drugs like paraquat and menadione [10], [11]. Subsequent studies revealed that in this microorganism SoxR mediates its effects in a two-step process. Upon sensing redox stress via its [2Fe-2S] clusters, SoxR activates the expression of a second transcription factor, *soxS* [12], [13]. SoxS, an AraC-type regulator then recruits RNA polymerase to the promoters of >100 genes (the SoxRS regulon), whose protein products cumulatively restore redox homeostasis and repair oxidant-induced cellular damage [14].

The *E. coli* SoxRS regulon is conserved in other enterobacteria where it functions to confer generalized protection against exogenous redox-cycling compounds. Various lines of research conducted in the past decade indicate that this function may be limited to members of the *Enterobacteriaceae,* and that the SoxR regulatory network is different in other bacteria. A comprehensive bioinformatic survey of sequenced bacterial genomes revealed that while a *soxR* homolog is detected in 176 genomes, a *soxS* homolog is present only in enteric bacteria where it appears to be the solitary gene directly regulated by SoxR [1]. The same study showed that in non-enterics (all of which lack *soxS*), SoxR is predicted to directly regulate a small number of genes. In further contrast to the apparent function of SoxR in enteric bacteria, none of the putative SoxR targets in non-enterics encode proteins that are typically involved in oxidative stress detoxification and repair. Instead these genes encode membrane transporters and enzymes with homology to proteins that modify small molecules, including antibiotics. The absence of a *soxS* homolog and the predicted SoxR regulons in non-enterics has given rise to the notion that SoxR does not regulate a generalized oxidative stress response in the majority of bacteria. This has been corroborated for *Pseudomonas putida, P. aeruginosa,* and *S. coelicolor,* where deletion of *soxR* does not result in increased sensitivity to redox-cycling drugs when compared to the parental strain [5], [9], [15], [16]. While the function of SoxR in *P. putida* remains unknown, SoxR regulates gene expression in response to redox-active endogenously produced antibiotics in both *P. aeruginosa* and *S. coelicolor,* indicating that SoxR plays a role that is intimately tied in with the physiology of these organisms [5], [6], [9], [17], [18].

The SoxR regulon in *P. aeruginosa* is induced during the production of phenazine antibiotics in stationary phase, and consists of two membrane transporters (encoded by *PA4205-PA4208* and *PA3718*) and a monooxygenase enzyme (*PA2274*) [15], [17]. The *S. coelicolor* SoxR regulon is also induced in stationary phase during the production of the benzochromanequinone blue-pigmented antibiotic actinorhodin (Act), and is similar to the *P. aeruginosa* regulon in encoding an ABC-type membrane transporter (*SCO7008*) and four redox enzymes (*SCO1178, SCO1909, SCO2478, SCO4266*) [5], [6]. It is noteworthy that two of these genes encode products that are similar to enzymes that catalyze tailoring steps in the Act biosynthetic pathway. Specifically, *SCO4266* is similar to the oxidoreductases ActVI-ORF2 (37% identity; 49% similarity) and ActVI-ORF4 (34% identity; 44% similarity), and *SCO1909* resembles the monooxygenase ActVA-ORF6 (38% identity; 55% similarity) [19]. *SCO1909* is also similar to *PA2274* (37% identity; 45% similarity), the SoxR-regulated monooxygenase in *P. aeruginosa* [19]. An independent study had also described four of the SoxR-targets in *S. coelicolor* as genes whose expression is temporally coordinated with Act (*eca*), and whose levels are reduced in an Act-non producing strain: *SCO7008 (ecaA), SCO1909 (ecaB), SCO1178 (ecaC), SCO4266 (ecaD)* [20]. The composition of the *P. aeruginosa* and *S. coelicolor* SoxR regulons, and the fact that they are induced by endogenously produced redox-active antibiotics, suggests that SoxR evolved to sense endogenous metabolites and activate machinery to process and transport them in these two phylogenetically divergent bacteria. This notion is further supported by the observation that both *P. aeruginosa* and *S. coelicolor soxR* mutants display de-regulated antibiotic production/secretion [1], [6].

Both the SoxR regulon in *P. aeruginosa,* and the SoxRS regulon in *E. coli* were characterized using microarray-based expression profiling [14], [15]. The five-gene SoxR regulon in *S. coelicolor,* on the other hand, was identified by conducting an *in silico* search of the genome for SoxR-binding sites located upstream of predicted open reading frames (ORFs) [1], [5], [6]. Given the morphological complexity and large genome size of *S. coelicolor* (8.7 megabases), it is possible that the bioinformatic screen for SoxR-regulated genes may have missed potential targets. This analysis would also have failed to identify genes that are indirectly regulated by SoxR (i.e. via an intermediate transcription factor as in *E. coli*). In this study we conducted a whole genome transcriptome comparison of the wild type *S. coelicolor* strain M145 and a *soxR* null mutant using RNA-Seq with the goal of gaining a comprehensive picture of the SoxR regulatory network in this medically important antibiotic-producer. This analysis revealed a sixth SoxR-regulated gene in *S. coelicolor,* in addition to the five confirmed targets.

## Materials and Methods

### Bacterial strains, culture conditions and RNA isolation

The *S. coelicolor* strains used in this study are listed in **Table 1**. For RNA-Seq and qRT-PCR experiments, about 10^8^ spores were cultured on R2YE plates [21] that were overlaid with cellophane and grown at 30°C for either 24 h (prior to the production of Act) or 72 h (when Act-proficient cells produce Act), with biological replicates obtained for the 72 h cultures. To harvest, cells were incubated with RNAprotect bacterial reagent (Qiagen) for 5 min at room temperature, scraped off the cellophane, pelleted by centrifugation for 10 min at 5,000×g, and frozen at −80°C. Cells were lysed by incubation for 15 min at 30°C in TE buffer containing 15 mg/mL lysozyme, followed by 30 s of sonication on ice. Total RNA was extracted with an RNeasy plant minikit (Qiagen) according to manufacturer’s instructions. Contaminating DNA was removed by treating for 1 h at 37°C with 5 units of RNase-free DNase I (Qiagen). The RNA preparation was subject to one extraction with acidified phenol-chloroform, followed by an extraction with chloroform-isoamyl alcohol. RNA was ethanol precipitated overnight at −80°C, washed with 80% ethanol and resuspended in nuclease-free water. RNA purity and concentration were determined using a Nanodrop or Qubit spectrophotometer. RNA quality was assessed by agarose gel electrophoresis or a Nano Bioanalyzer. The absence of contaminating DNA was confirmed by the absence of product following a 30-cycle PCR reaction using RNA as template and *hrdB* primers (**Table S1**).

**Table 1:** *Streptomyces coelicolor* strains used in this study.

Strain	Genotype or description	Reference
M145	SCP1^−^ SCP2^−^ derivative of A3(2)	[21]
M511	Δ*act*II-ORF4 derivative of M145	[32]
M145-1A	Markerless Δ*soxR* derivative of M145	[5]
145/pSET152	M145 transformed with pSET152	[5]
Δ*soxR*/pSET152	M145-1A transformed with pSET152	[5]
Δ*soxR*/pSoxR	M145-1A transformed with pSET152::*soxR*	[5]

### Library construction and RNA sequencing

Library construction and sequencing of RNA transcripts was performed by Fasteris SA (Switzerland). Briefly, RNA samples were treated to reduce ribosomal RNA levels using the Ambion MICROBExpress kit. RNA transcripts were fragmented using a buffered zinc solution. The first cDNA strand was synthesized by reverse transcription using random primers in the presence of dUTPs. After second strand synthesis and adapter ligation, the first cDNA strand was digested with uracil-DNA glycosylase. The remaining fragments were PCR amplified and 150–200 bp amplicons were selected by polyacrylamide gel electrophoresis. The resulting cDNAs underwent high-throughput sequencing in an Illumina HiSeq 2000 sequencer to obtain single reads of 100 bp using the forward sequencing primer.

### Read mapping and differential expression

The sequenced reads were mapped on to the *S. coelicolor* genome [22] using BWA v0.5.9 [23] and read counts for each annotated transcript were compiled using BEDtools without regard to strand [24], on the web-based platform Galaxy [25]–[27]. For both the wild type and Δ*soxR* libraries, ∼97% of total reads were mapped to the *S. coelicolor* genome. Differential gene expression was assessed using DESeq with library sizes normalized by the median count ratios across transcripts and false discovery rate adjusted q-values calculated according to the Benjamini-Hochberg procedure [28]. The data supporting the results of this work are available in NCBI’s Gene Expression Omnibus and accessible through GEO series accession number GSE57268 (http://www.ncbi.nlm.nih.gov/geo/query/acc.cgi?acc=GSE57268).

### Re-annotation of SCO0319, SCO0320, SCO0321 region

To identify alternative potential transcripts in the region surrounding *SCO0319*- *SCO0321* the *S. coelicolor* genome region spanning *SCO0318* to *SCO0321* (318329–322255) was scanned for open reading frames (ORFs) using Geneious (v.7.1.4, Biomatters). Large ORFs (>500 bp) that spanned the region of *SCO0319* to *SCO0320* were selected and a BLAST search [29] was performed to identify related sequences and elucidate potential gene function.

### Quantitative RT-PCR

The cDNA templates for qRT-PCR were generated from total RNA with iScript (Bio-Rad). The primers used for qRT-PCR (Integrated DNA Technologies) were designed using Primer3 software [30], with a melting temperature of 60°C, length of ∼20- nt, and amplicon length of ∼100 bp (**Table S1**). Each qRT-PCR reaction (20 µL) contained 25 ng cDNA, 250 nM each of forward and reverse primer, and 10 µL Power Sybr green PCR master mix (Applied Biosystems). qRT-PCR reactions were carried out in a StepOne PCR machine (Applied Biosystems) with the following reaction parameters: 10 min at 95°C; 40 two-step amplification cycles with 15 s denaturation at 95°C and 1 min annealing and extension at 60°C; final dissociation stage for 15 min to generate a melting curve and verify specificity of amplification products. Samples were assayed in duplicate and the target signal standardized to the level of the housekeeping sigma factor *hrdB*.

### Electrophoretic mobility shift assay (EMSA)

A gel mobility shift assay was used to assess the binding of purified SoxR to promoter DNA. The method of Dela Cruz *et al.* [5] was used to purify histidine-tagged SoxR and generate DIG-labeled DNA probes. Primers used to PCR amplify the promoter DNA fragments are listed in **Table S1**. For the binding reaction, 6 fmol of DNA probe was incubated with SoxR (0 to 20 nM) for 15 min at 25°C in binding buffer (10 mM Tris-HCl, pH 8, 75 mM KCl, 0.1 mM dithiothreitol, 10% glycerol, 2 mM MgCl_2_, 0.1 µg poly(dI)-poly(dC), 2 mM dAMP) in a total volume of 30 µL. For the competition assay, 3,000 fmol of unlabeled probe (specific competitor) was added to the reaction mixtures. Protein-bound and uncomplexed DNA products were separated on a 5% polyacrylamide gel (Tris-HCl, pH 8, 3.3 mM sodium acetate, pH 7.9, 1 mM EDTA, 2% glycerol) that was run at 6°C and 180 V for 90 min. The DNA was transferred to nylon membranes (Roche), cross linked by UV, and detected using a DIG gel shift 2^nd^-generation kit (Roche) according to manufacturer’s instructions.

### Reverse Transcription PCR (RT-PCR)

RT-PCR reactions were carried out on total RNA using the OneStep RT-PCR kit (Qiagen) according to manufacturer’s instructions. All RT-PCR reactions consisted of 30 cycles using primers listed in **Table S1**. Reactions were analyzed on TAE: agarose gels and nucleic acids were visualized using ethidium bromide.

### Bioinformatic analysis of Streptomyces genomes for potential SoxR-regulated genes

Whole genome sequences of *Streptomyces* species downloaded from the NCBI genomes database (ftp://ftp.ncbi.nih.gov/genomes/) were searched for potential homologs of the genes of the *Streptomyces coelicolor* SoxR regulon using tblastn [29], retaining only the best matching genomic region for each gene. If two of the regulon genes matched to the same region of the genome, only the best matching gene was reported. For each retained matching sequence, the region 1kb upstream was searched for the soxbox binding motif [1] using a position-specific scoring matrix applied via the BioPython toolkit [31]. The nucleotide frequencies for each target genome were used to calculate expected background match rates, and matches with a log-odds score for the soxbox motif greater than 10 are reported.

## Results

### RNA-Seq analysis reveals several novel genes as potential SoxR targets

The previously described five-gene SoxR regulon in *S. coelicolor* was identified bioinformatically [5], [6]. In order to identify other potential SoxR-regulated genes that might have been missed by this bioinformatic approach, we conducted RNA-Seq analysis to detect genes differentially expressed between wild type and a Δ*soxR* mutant strain. RNA substrates were extracted from hyphae grown on R2YE medium. Because Act (or a precursor) is a known physiological activator of SoxR in *S. coelicolor* [5], [6], samples were collected three days post-inoculation when cells were actively producing this blue-pigmented antibiotic. As a control, samples were also collected 24 h post-inoculation, when no pigmented antibiotics were visible (and SoxR is quiescent). To identify genes that were potentially both SoxR- and Act- dependent, we focused on those that met two criteria: (i) were differentially expressed between wild type and Δ*soxR* in 3-day old cultures (Act produced); (ii) were also differentially expressed between 3-day old (blue) and 1-day old (unpigmented) wild type cultures. As predicted, the five established members of the SoxR regulon (*ecaA-ecaD, SCO2478*) were all significantly overexpressed in wild type compared to the Δ*soxR* mutant (**Table 2**). Each showed a ≥4-fold differential expression in wild type versus Δ*soxR*, and a q-value of ≤0.5. Using these parameters (≥4-fold differential expression and a q-value of ≤0.5), twelve novel genes were discovered to be potentially up-regulated by SoxR in stationary phase (**Table 2**). Of these twelve genes, three (*SCO7688, SCO7682, SCO2878*) did not display differential expression between 1-day and 3-day old wild type cultures (**Table 2**). These genes are thus unlikely to be Act-dependent and were eliminated from further analysis.

**Table 2:** Genes identified by RNA-Seq as SoxR-dependent in stationary phase^a^.

*SCO* number	Decrease in Δ*soxR* versus WT on Day 3^b^		Increase in WT on Day 3 vs. Day1^c^		Predicted function^e^
	Fold change	q-value^d^	Fold Change	q-value^d^	
*0319*	84	2e-28	>100	9e-12	Hypothetical protein
*0320*	79	3e-21	52	5e-10	Quinone oxidoreductase
*1178* f *(ecaC)*	52	2e-21	49	1e-9	NAD-dependent epimerase/dehydratase
*4266* f *(ecaD)*	30	2e-28	14	4e-7	Oxidoreductase
*7688* g	28	8e-2	1	1e0	Hypothetical protein
*1177*	23	7e-18	32	2e-7	GntR-family transcriptional regulator
*0321*	18	7e-18	26	9e-8	Carboxylesterase
*1909* f *(ecaB)*	18	1e-22	11	7e-6	Monooxygenase
*1734*	15	4e-11	24	2e-5	Secreted cellulose binding protein
*4021*	11	6e-2	2	8e-1	Two-component histidine kinase
*2478* f	7	3e-1	>100	1e-3	Flavoprotein reductase
*7682* g	6	4e-1	<1	8e-1	Non-ribosomal peptide synthase
*4157*	6	4e-1	4	4e-1	Protease
*4020*	6	3e-3	2	8e-1	Two-component response regulator
*6165*	6	3e-1	>100	8e-4	Hypothetical protein
*1697* h	5	8e-3	1	1e0	SoxR
*2878* g	5	4e-1	<1	5e-1	Hypothetical protein
*7008* f *(ecaA)*	5	3e-9	37	2e-9	ABC transporter

a Genes are organized in decreasing order of SoxR-dependence as determined by RNA-Seq.

b RNA for this comparison was obtained from 3-day old WT or Δ*soxR* cultures, both of which were blue-pigmented.

c RNA for this comparison was obtained from 3-day old WT (blue-pigmented) or 1-day old WT (unpigmented) cultures.

d False discovery rate adjusted q-values were calculated according to the Benjamini-Hochberg procedure [27].

e Predicted functions of genes were obtained from StrepDB (http://strepdb.Streptomyces.org.uk).

f Confirmed SoxR-targets that are directly regulated by SoxR in response to Act production [5], [6].

g These genes are unlikely to be Act-dependent since they were not differentially expressed between 3-day old versus 1-day old WT samples, and were not considered for further analysis.

h *SCO1697* is *soxR* which is constitutively expressed over the course of development, and is not autoregulated [5].

### Quantitative real-time PCR confirms SoxR- and Act-dependence in a subset of genes identified by RNA-Seq

To validate the RNA-Seq results, the SoxR-dependence of the nine newly identified genes was analyzed by quantitative real time PCR (qRT-PCR). RNA was obtained from independent biological samples (wild type and Δ*soxR*) following growth for three days on complex agar (R2YE) medium. Six of the nine newly identified genes were significantly overexpressed in wild type as compared to Δ*soxR* (≥4-fold difference) by qRT-PCR analysis: *SCO0319, SCO0320, SCO0321, SCO1177, SCO1734, SCO4021* (**Table 3**). The other three genes that were tagged as SoxR-dependent by RNA-Seq (*SCO4020, SCO4157, SCO6165*) did not demonstrate differential expression between wild type and Δ*soxR* in the qRT-PCR assay and were eliminated from further analysis (**Table 3**).

**Table 3:** Validation of RNA-Seq results by quantitative RT-PCR^a^.

*SCO* Number	Fold decrease in Δ*soxR* versus WT^b^	Fold decrease in Δ*act* versus WT^c^	Predicted function
Potential SoxR-targets^d^			
*0319*	4	24	Hypothetical protein
*0320*	86	34	Quinone oxidoreductase
*0321*	59	61	Carboxylesterase
*1177*	9	11	GntR-family transcriptional regulator
*1734*	8	3	Secreted cellulose binding protein
*4020* e	1	1	Two-component response regulator
*4021*	10	2	Two-component histidine kinase
*4157* e	1	2	Protease
*6165* e	1	<1	Hypothetical protein
*soxR* and confirmed SoxR-targets			
*1697*	47	1	SoxR
*1178 (ecaC)*	60	29	NAD-dependent epimerase/dehydratase
*1909 (ecaB)*	30	30	Monooxygenase
*2478*	31	15	Flavoprotein reductase
*4266 (ecaD)*	29	59	Oxidoreductase
*7008 (ecaA)*	15	30	ABC transporter

a RNA for qRT-PCR validation was obtained from independent biological samples.

b Differential gene expression in WT and Δ*soxR* in 3-day old cultures assessed by qRT-PCR. Gene expression was standardized to the housekeeping sigma factor, *hrdB,* and normalized to WT.

c Differential gene expression in WT and the Δ*act* strain (M511) in 3-day old cultures assessed by qRT-PCR. Gene expression was standardized to the housekeeping sigma factor, *hrdB,* and normalized to WT.

d Genes are arranged in order of increasing *SCO* number.

e These genes did not demonstrate SoxR- or Act-dependent expression in the qRT-PCR validation assay and were eliminated from further analysis.

Since SoxR is transcriptionally active only in Act-producing cells, SoxR-dependent genes should also demonstrate Act-dependence. This is true for the five previously confirmed SoxR targets (**Table 3**). To determine if the six new candidate SoxR-targets demonstrate Act-dependence, their expression levels were compared in 3-day old wild type and the Act-deficient strain M511 (a strain with an in-frame deletion of the pathway-specific regulator of Act biosynthesis, *actII*–ORF4) [32]. Four of the six new candidate SoxR targets (*SCO0319, SCO0320, SCO0321,* and *SCO1177)* were significantly under-expressed (≥11-fold difference) in M511 compared with wild type, while *SCO1734* and *SCO4021* showed a more modest (2–3 fold) difference in expression levels between the two backgrounds (**Table 3**).

**Figure 1:**
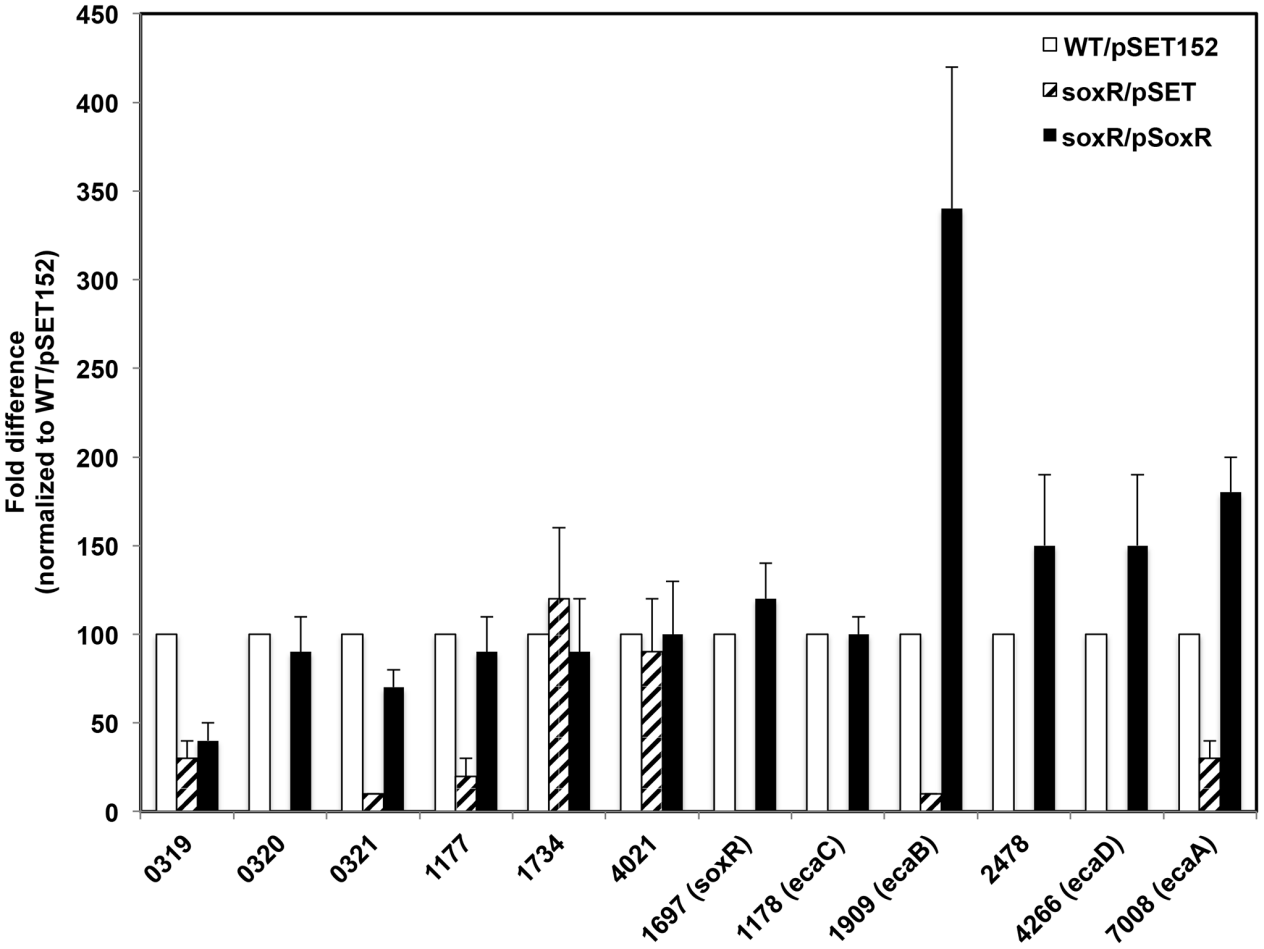
Complementation analysis to confirm SoxR-dependence of genes identified by RNA-Seq. qRT-PCR was performed on RNA isolated from WT/pSET152, Δ*soxR*/pSET152, and a Δ*soxR* strain complemented with wild-type *soxR* (pSoxR), that were grown on R2YE plates for 3 days. The expression levels of all genes were standardized to the level of the constitutively expressed housekeeping sigma factor, *hrdB,* and normalized to expression in WT/pSET152. The results represent the means and standard deviation (bars; some are not visible on this scale) of four independent experiments.

To further confirm that reduced expression of the six new potential SoxR-targets in the Δ*soxR* background was due to SoxR-deficiency, we conducted a complementation experiment. Using qRT-PCR, the expression levels of the relevant genes (along with known controls) were analyzed in the complemented Δ*soxR* strain that expresses *soxR* from a chromosomally integrated plasmid pSET152 (**Table 1**). **Figure 1** shows that expression of *SCO1697 (soxR),* and its five confirmed targets (*ecaA-ecaD, SCO2478*) was restored in the *soxR* null strain complemented with a wild type copy of *soxR* (soxR/pSoxR). Furthermore, expression of three genes that showed significant Act-dependence (**Table 3**), *SCO0320, SCO0321* and *SCO1177*, was also rescued in the *soxR-*complemented strain (**Figure 1**). By contrast, *SCO0319, SCO1734* and *SCO4021* failed the complementation test. In this experiment, *SCO0319* showed similar expression levels in the Δ*soxR* and the complemented Δ*soxR* backgrounds, while *SCO1734* and *SCO4021* were similarly expressed in wild type, Δ*soxR* and the complemented Δ*soxR* backgrounds (**Figure 1**). Given these results *SCO0319, SCO1734* and *SCO4021* were eliminated as SoxR-targets.

In summary, RNA-Seq analysis followed by qRT-PCR validation identified eight genes that are both SoxR- and Act-dependent. Five of these are previously confirmed members of the *S. coelicolor* SoxR regulon [5], [6]. Of the newly identified SoxR-induced genes, it appeared that *SCO0320* (homologous to the carboxy-terminal half of *SLI_0274,* a quinone oxidoreductase in *Streptomyces lividans* ***,*** **Figure S2**) and *SCO0321* (carboxylesterase) may be transcriptionally coupled. As redox-associated enzymes, *SCO0320* and *SCO0321* functionally cluster with the known SoxR-targets, *ecaB, ecaC, ecaD and SCO2478*. *SCO1177* encodes a putative GntR-family transcriptional regulator. The GntR family members (so named for the *Bacillus subtilis* repressor of the gluconate operon) normally act as transcriptional repressors, and regulate gene expression in response to nutritional and/or other environmental signals [33].

### SoxR binds to the promoter of only one of its new putative targets (with a twist)

As mentioned before, SoxR homologs from different bacteria have highly conserved DNA binding domains, and thus bind to similar operator sequences (soxbox) in the promoters of their target genes. We previously demonstrated that SoxR directly binds to the promoters of its five known targets in *S. coelicolor* (all of which share a similar promoter architecture) to directly activate their transcription [5]. However, visual inspection of the DNA region upstream of the newly identified potential SoxR targets, *SCO0320, SCO0321,* and *SCO1177* failed to reveal potential SoxR docking sites. Nevertheless, we decided to empirically determine if SoxR binds to the promoter regions of its three new putative target genes by electrophoretic mobility shift assays (EMSA). Towards this end we incubated purified SoxR with DIG-end-labeled DNA fragments that span ∼170–200 bp upstream of each predicted ORF. While these assays showed that SoxR bound at the expected dissociation constant of 5 nM to the promoter of *ecaD* (a known target), we were unable to detect binding to any of the DNA probes for the new potential targets, even at the highest concentration tested (20 nM; data not shown).

**Figure 2:**
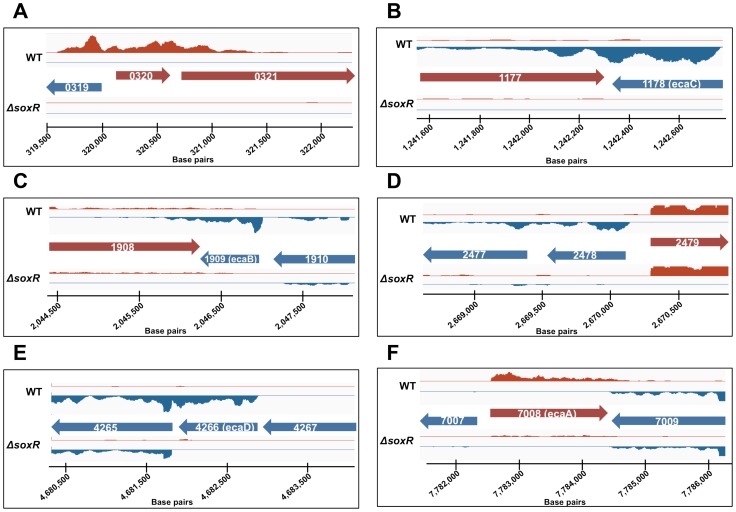
RNA-Seq reads visualized on Integrative Genomics Viewer. Reads were obtained from RNA isolated from 3-day old wild type and Δ*soxR* samples grown on R2YE medium. Red indicates the positive strand; blue indicates the negative strand; scale bar indicates the chromosomal position in the *S. coelicolor* M145 genome. A) *SCO0319-SCO0321;* B) *SCO1177-SCO1179;* C) *SCO1908-SCO1910;* D) *SCO2477-SCO2479;* E) *SCO4265-SCO4267;* F) *SCO7007-SCO7009.*

**Figure 3:**
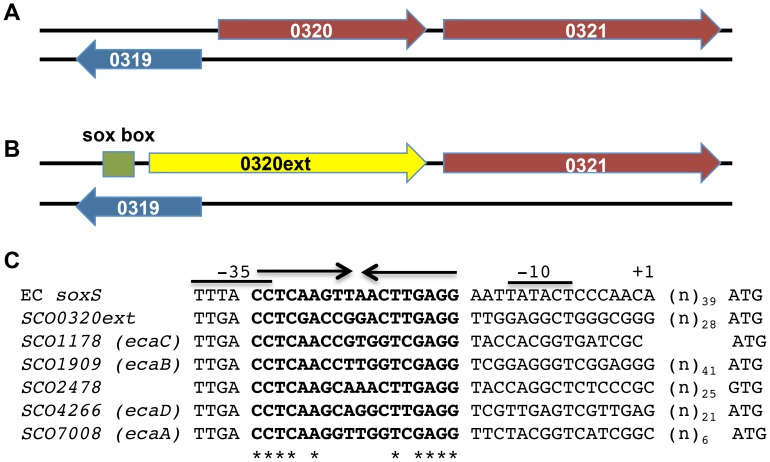
Reannotation of the *SCO0319-SCO0321* region in *S. coelicolor*. (A) *SCO0319* is annotated in StrepDB as transcribed divergently from *SCO0320* and *SCO0321*. (B) Reannotation of the same region using Geneious (v.7.1.4 Biomatters) predicts that *SCO0320* initiates further upstream within the *SCO0319* ORF; the reannotated ORF is renamed *SCO0320ext.* A conserved SoxR-binding site (soxbox) is positioned upstream of *SCO0320ext.* (C) The putative promoter of *SCO0320ext* is aligned with the *E. coli soxS* promoter and the promoters of five confirmed SoxR target genes in *S. coelicolor.* SoxR binding sites are indicated in bold type, and the inverted arrows depict the sequence of dyad symmetry. The asterisks indicate conserved nucleotides within the SoxR binding site. The transcriptional start site of *E. coli soxS* is labeled +1, and the −10 and −35 sequences are indicated. The number of nucleotides to the predicted start codons of the different genes is shown.

Given these puzzling findings, we examined the RNA-Seq reads for all SoxR-regulon members more closely on Integrative Genomics Viewer (**Figure 2**) [34], [35]. The five known SoxR targets (*ecaA-ecaD, SCO2478*) appeared as expected, displaying strong expression on the annotated DNA strand in the wild type background, and very low (to negligible) reads in the Δ*soxR* background (**Figure 2B–F**). An examination of the RNA-Seq reads for the *SCO0319-SCO0321* region, however, revealed a few unexpected features. According to the annotation in StrepDB (http://strepdb.Streptomyces.org.uk), *SCO0320* and *SCO0321* are transcribed from the same strand, while *SCO0319* is divergently transcribed from the complementary strand (**Figure 2A** **and** **3A**). However, the RNA-Seq reads for all three genes mapped on the same strand, and furthermore, large numbers of reads mapped to the intergenic regions between the three genes (**Figure 2A**). To investigate the possibility that these reads derived from a previously unannotated transcript, we reannotated and manually curated all possible ORFs within this region. An alternate ORF that spans the region of what is currently annotated as *SCO0319* and *SCO0320* was identified, which we have named *SCO0320ext* (corresponding to chromosomal positions 319614-320569; **Figure 3B**). A BLAST search [29] of the translated amino acid sequence from *SCO0320ext* revealed that its N-terminal region is homologous to *SLI_0274*, a quinone oxidoreductase in *S. lividans,* the C-terminus of which had previously been identified as homologous to *SCO0320* (**Figure S2**). Intriguingly, the DNA region upstream of *SCO0320ext* revealed a potential SoxR binding site and a promoter architecture that resembles the promoters of the five confirmed SoxR targets in *S. coelicolor* (**Figure 3C**).

**Figure 4:**
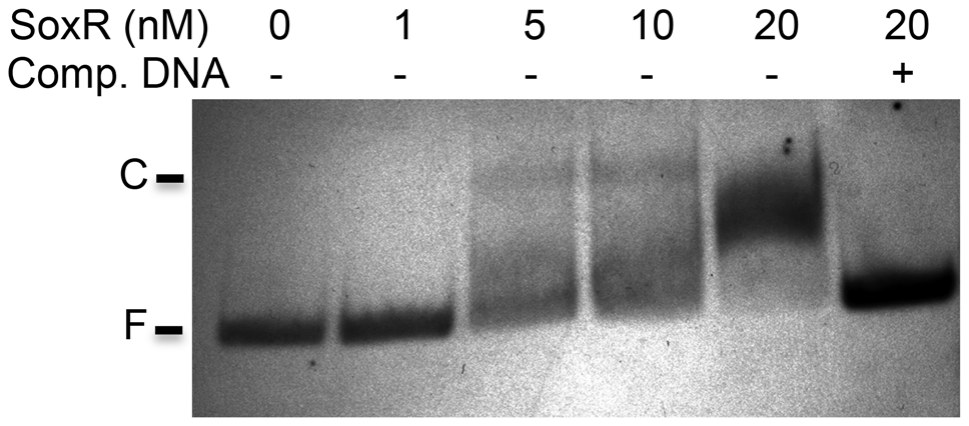
SoxR binds to the promoter of *SCO0320ext in vitro*. A DIG-end-labeled DNA fragment spanning the putative promoter region of *SCO0320ext* was incubated with increasing amounts of purified histidine-tagged SoxR protein. Protein-bound complexes (C) and free DNA (F) were separated on a 5% native polyacrylamide gel. The specificity of SoxR binding was demonstrated by the addition of a 500-fold molar excess of unlabeled competitor probe.

To test if SoxR binds to the putative promoter of *SCO0320ext*, we conducted EMSA with purified SoxR and a DNA fragment that spans the region upstream of *SCO0320ext*. **Figure 4** shows that SoxR binds to this region with high affinity; the amount of protein needed to bind 50% of the DNA was between 5 and 10 nM (**Figure 4**), which is comparable to the affinity of SoxR for its other target genes in *S. coelicolor* [5]. Specificity of binding was demonstrated by addition of 500-fold excess of unlabeled competitive DNA that resulted in displacement of SoxR from the labeled probe (**Figure 4**). This result, combined with the qRT-PCR assays described above, strongly suggests that *SCO0320ext* is a direct SoxR target, and the sixth member of the SoxR regulon in *S. coelicolor*.

### SCO0321 and SCO1177 are not SoxR targets, but artifacts of transcriptional read-through

**Figure 5:**
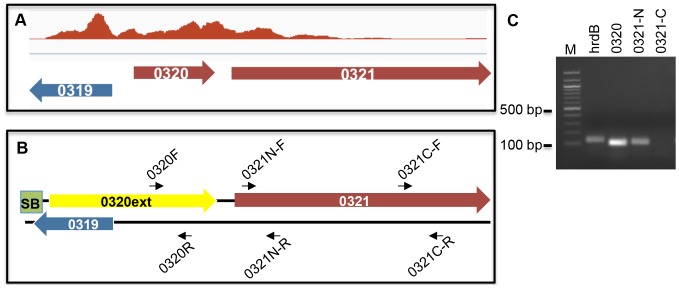
Transcription of *SCO0320ext* results in read-through into the 5′ region of *SCO0321*. (A) RNA-seq reads of the *SCO0319-SCO0321* region obtained from RNA obtained from three-day old M145 wild type cells. (B) Schematic representation of reannotated *SCO0319-SCO0321* region. Predicted ORFs are indicated by block arrows; the box labeled “SB” indicates the SoxR-binding site upstream of *SCO0320ext.* The primers used for RT-PCR analysis are indicated; primer sequences are reported in Table S1. (C) RT-PCR analysis was carried out on RNA isolated from 3-day old wild type cultures grown on R2YE plates. The *hrdB* gene encodes the house-keeping sigma factor, and was included as a control. *SCO0320ext* was amplified using primers 0320F and 0320R; the 5′ region of *SCO0321* was amplified with primers 0321N-F and 0321N-R; the 3′ region of *SCO0321* was amplified with primers 0321C-F and 0321C-R. The first lane contains the 100-bp marker (New England Biolabs).

These results helped explain how SoxR regulates transcription of *SCO0320ext.* However, we were left with two other potential SoxR targets (*SCO0321* and *SCO1177)* that do not appear to harbor a SoxR-binding site in their putative promoters, leaving the mechanism of their regulation unclear. Given that the RNA-seq reads for *SCO0321* are concentrated within the 5′ end of the gene (**Figures 2A** **and** **5A** **)**, we propose that *SCO0321* is not a real SoxR-target, but likely an artifact of transcriptional read-through from *SCO0320ext*. The primers that were used in the previously described qRT-PCR validation assays for *SCO0321* (0321N-F and 0321N-R; **Figure 5B**) bind within the 5′ region of the gene that demonstrated high RNA-Seq reads (**Figure 5A**). However, when we used a different set of primers (0321C-F and 0321C-R; **Figure 5B**) that bind within the 3′ region of the gene, we did not observe an RT product (**Figure 5C**). This result is consistent with the lack of RNA-seq reads within the 3′ region of *SCO0321* and indicates that the complete sequence of *SCO0321* is not independently transcribed under our experimental conditions.

An examination of the RNA-Seq reads for *SCO1177* similarly suggested that this gene is not a SoxR-target but an artifact of transcriptional read-through from the adjacent gene, *SCO1178 (ecaC),* a confirmed SoxR regulated gene. As annotated in StrepDB, *SCO1177* and *ecaC* are convergently transcribed from opposite strands, however all RNA-Seq reads for *SCO1177* map to the sense strand for *ecaC* (**Figure 2B**). Moreover, the reads for *SCO1177* cluster towards the 3′ end of the gene that is closest to *ecaC,* and could readily be explained by transcriptional read-through of *ecaC.* Here, *SCO1177* was detected as a SoxR-target in our initial RNA-Seq analysis because read counts were compiled without regard to strand specificity. Furthermore, the primers used in the qRT-PCR validation assays for *SCO1177* happen to prime within the 3′ region of this gene that demonstrated high RNA-Seq reads (**Figure 2B**). It should be noted that we did not observe significant reads in the region corresponding to the sense strand of *SCO1177* in either wild type or Δ*soxR* strains at any of the time points sampled (day 3 reads shown in **Figure 2B**; day 1 data not shown). Thus, *SCO1177* also does not appear to be expressed under the growth conditions used in this work.

## Discussion

In this work we expanded the SoxR regulon in *S. coelicolor* by comparing the transcriptomes of wild type and *soxR* null mutant strains in stationary phase using RNA-Seq. This regulon is composed of six genes induced by SoxR in response to the redox-active antibiotic Act that is produced in stationary phase. Five of these genes (*ecaA-ecaD, SCO2478*) were previously identified as SoxR-dependent using a bioinformatics approach designed to identify genes based on the presence of a SoxR-binding site in putative promoter regions [5], [6]. The Act-dependent expression of four of the SoxR-targets (ecaA-ecaD) was independently described by Huang and co-workers [20]. The sixth S. coelicolor SoxR-regulon member identified in this work, *SCO0320ext,* was missed by the bioinformatic search for SoxR-targets, and was also not previously identified as an “*eca”* gene.

*SCO0320ext* encodes a putative quinone oxidoreductase with homology to *SLI_0274 *in *S. lividans* and *SAV_4018 *in *Streptomyces avermitilis* (**Table 4**). Both the *S. lividans* and *S. avermitilis* homologs of *SCO0320ext* contain putative SoxR-binding sites in their promoters suggesting that they may be under SoxR regulation in these organisms as well. It is peculiar that only the amino-terminal half of *SCO0320ext* is homologous to the other two proteins. As illustrated in **Figure S2**, there is almost complete sequence identity between the amino-terminal halves of *SCO0320ext* and *SLI_0274*, but clear divergence in the carboxy-terminal halves. An alignment of the nucleotide sequences of *SLI_0274* and *SCO0320ext* shows a single base difference (deletion of cytosine 494) in *SCO0320ext* that causes a shift in the open reading frame (**Figure S2A**). Interestingly, *SCO0320* (as annotated in StrepDB) is almost identical to the carboxy-terminal half of *SLI_0274* (**Figure S2**). Thus it appears that *S. coelicolor* M145 acquired a mutation making what was originally one gene (that corresponding to *SCO0320ext*), appear to be two separate genes (*SCO0319* and *SCO0320*). Analysis of the nucleotide sequence of this region in two other *S. coelicolor* A3(2) derivatives (M600 and J1501) showed the same base deletion found in M145 (data not shown). At this point it is unclear if *SCO0320ext* is functional in *S. coelicolor*, or if the mutation has any physiological consequences for the organism.

**Table 4:** Conservation of the *S. coelicolor* SoxR regulon in other *Streptomyces* species^a^.

Predicted function		*S. coelicolor* homolog in^b^:					
	*SCO*	*SAV* c	*SCLAV*	*SGR*	*SLI*	*SCAB*	*SVEN*
Quinone oxidoreductase	*0320ext*	*4018*	None	None	*0274*	None	None
Monooxygenase	*1909 (ecaB)*	None	*1111*	*5610*	*2221*	*70221*	*1541*
NAD-dependent epimerase/dehydratase	*1178 (ecaC)*	None	*p0595*	*3374*	*1455*	*5581*	*4172*
Flavoprotein reductase	*2478*	*5665*	*1679*	*5059*	*2813*	*62351*	*2266*
Oxidoreductase	*4266 (ecaD)*	*3956*	*3239*	*4037*	*4501*	None	*4021*
ABC transporter	*7008 (ecaA)*	*7218*	*0059*	*6589*	*7210*	*12171*	None

a *Streptomyces* species listed are as follows: *SCO, S. coelicolor; SAV, S. avermitilis; SCLAV, S. clavuligerus; SGR, S. griseus; SLI, S. lividans; SCAB, S. scabies; SVEN, S. venezuelae*.

b Each gene listed in this table contains a putative SoxR-binding site within 200 bp upstream of its predicted open reading frame in each species in which it was found. Sequences are available in Table S4.

c The *S. avermitilis* genome harbors two other genes whose promoters contain conserved SoxR binding sites: *SAV_1623* (putative transketolase, and *SAV_4017* (putative TetR family transcriptional regulator).

SoxR is typically considered a transcriptional activator. Nevertheless, we analyzed our RNA-Seq data for genes whose expression may be inhibited by SoxR. Several candidate genes were found to be overexpressed in the Δ*soxR* mutant compared to wild type (≥4-fold difference; **Table S2**). From this list, thirteen genes showed statistically significant differential expression (q-value ≤ 0.1), and were selected for validation by qRT-PCR performed on RNA isolated from independent biological samples. Of these, only six showed significantly different expression levels (≥4-fold) in wild type and the Δ*soxR* mutant by qRT-PCR (**Table S3**). However, none of these six genes passed the complementation test, where we expected lower expression in the wild type and *soxR*-complemented backgrounds compared to the *soxR*–deficient background. Instead, most were overexpressed in the *soxR*-complemented strain contrary to the predicted pattern (**Figure S1**). It can thus be concluded that SoxR does not function as a transcriptional inhibitor in *S. coelicolor*.

**Figure 6:**
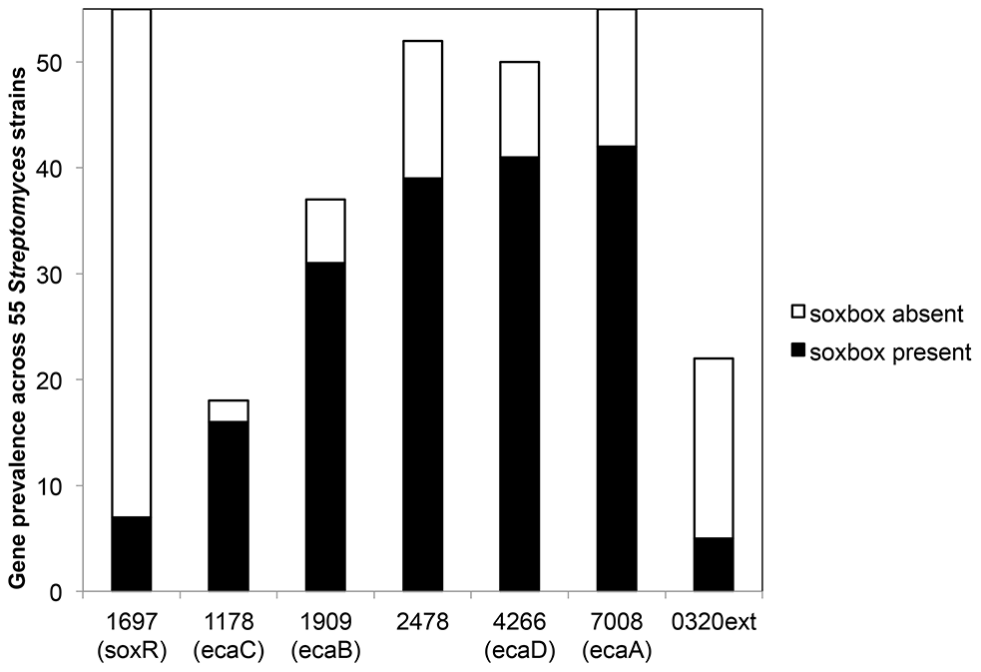
Extent of conservation of the SoxR regulon in 55 *Streptomyces* species. The graph shows the number of *Streptomyces* species surveyed that contain sequences homologous to *soxR* or SoxR-regulated genes from *S. coelicolor.* The black segment indicates the number of homologs with a soxbox motif within 1 kb upstream, while the white segment shows those that lack a soxbox sequence.

As described earlier, SoxR stimulates the production of a similar group of genes (transporters and redox enzymes) in *S. coelicolor* and *P. aeruginosa* in response to endogenously produced antibiotics; Act in the former and phenazines in the latter. This scenario appears to be maintained in other sequenced streptomycetes. An analysis of seven *Streptomyces* species that are annotated in the *Streptomyces* database (http://strepdb.Streptomyces.org.uk) shows that all six SoxR-regulated genes in *S. coelicolor* have homologs in its closest relative *S. lividans,* while a subset of these genes are conserved in the five other *Streptomyces* species (**Table 4**). Notably, all the genes listed in **Table 4** contain a SoxR-binding sequence (soxbox) in their promoters suggesting that they are under SoxR control (soxbox sequences available in **Table S4**). A more extensive survey of 55 sequenced *Streptomyces* species showed the presence of *soxR* in every species analyzed (**Table S4;** **Figure 6**). Interestingly, while *soxR* is not autoregulated in *S. coelicolor,* about 13 percent of *soxR* homologs in other *Streptomyces* surveyed showed a potential soxbox upstream suggesting that these homologs may be autoregulated. The suite of SoxR-regulated genes in *S. coelicolor* is fairly well conserved in the other streptomycetes analyzed, ranging from 100 percent conservation of *ecaA* to 33 percent for *ecaC* (**Table S4;** **Figure** 6). When found, these genes commonly contain a potential soxbox within one kilobase pairs upstream (**Table S4;** **Figure 6**). *SCO0320ext* is a notable exception with only 23 percent of homologs harboring a soxbox sequence. While none of the other species aside from *S. coelicolor* produce Act *per se*, members of this genus are known to produce other biologically active secondary metabolites. These molecules could serve as signals that trigger SoxR activity in other streptomycetes. Knowledge of the individual genes regulated by SoxR will facilitate further studies to elucidate the function of this regulatory protein and its regulon in antibiotic producers.

## Supporting Information

Figure S1 **Complementation analysis to further assess the SoxR-dependence of genes identified by RNA-Seq.**
(DOCX)

Figure S2 **Sequence alignment of** ***SCO0320ext*** **and** ***SCO0320*** **with the quinone oxidoreductase** ***SLI_0274*** **from** ***S. lividans.***
(DOCX)

Table S1 **Primers used in this study.**
(DOCX)

Table S2 **Genes identified by RNA-Seq as upregulated in Δ** ***soxR*** **compared to WT in stationary phase.**
(DOCX)

Table S3 **Validation of RNA-Seq data reported in Table S2 by quantitative RT-PCR.**
(DOCX)

Table S4 **tblastn matches of genes of the SoxR regulon to** ***Streptomyces*** **whole genome sequences regions.** Soxbox positions are listed relative to the 5′ end of the blast hit.
(XLSX)

## References

[1] Dietrich LEP, Teal TK, Price-Whelan A, Newman DK (2008) Redox-active antibiotics control gene expression and community behavior in divergent bacteria. Science 321: 1203–1206 10.1126/science.1160619 18755976 PMC2745639

[2] Nunoshiba T, Hidalgo E, Amábile-Cuevas CF, Demple B (1992) Two-stage control of an oxidative stress regulon: the *Escherichia coli* SoxR protein triggers redox-inducible expression of the soxS regulatory gene. J Bacteriol 174: 6054–6060. 1400156 10.1128/jb.174.19.6054-6060.1992 PMC207670

[3] Kobayashi K, Tagawa S (2004) Activation of SoxR-dependent transcription in *Pseudomonas aeruginosa* . J Biochem 136: 607–615 10.1093/jb/mvh168 15632300

[4] Eiamphungporn W, Charoenlap N, Vattanaviboon P, Mongkolsuk S (2006) *Agrobacterium tumefaciens* soxR is involved in superoxide stress protection and also directly regulates superoxide-inducible expression of itself and a target gene. J Bacteriol 188: 8669–8673 10.1128/JB.00856-06 17041041 PMC1698218

[5] Dela Cruz R, Gao Y, Penumetcha S, Sheplock R, Weng K, et al (2010) Expression of the *Streptomyces coelicolor* SoxR regulon is intimately linked with actinorhodin production. J Bacteriol 192: 6428–6438 10.1128/JB.00916-10 20952574 PMC3008532

[6] Shin J-H, Singh AK, Cheon D-J, Roe J-H (2011) Activation of the SoxR regulon in Streptomyces coelicolor by the extracellular form of the pigmented antibiotic actinorhodin. J Bacteriol 193: 75–81 10.1128/JB.00965-10 21037009 PMC3019960

[7] Mahavihakanont A, Charoenlap N, Namchaiw P, Eiamphungporn W, Chattrakarn S, et al (2012) Novel roles of SoxR, a transcriptional regulator from *Xanthomonas campestris*, in sensing redox-cycling drugs and regulating a protective gene that have overall implications for bacterial stress physiology and virulence on a host plant. J Bacteriol 194: 209–217 10.1128/JB.05603-11 22056938 PMC3256661

[8] Bradley TM, Hidalgo E, Leautaud V, Ding H, Demple B (1997) Cysteine-to-alanine replacements in the *Escherichia coli* SoxR protein and the role of the [2Fe-2S] centers in transcriptional activation. Nuc Acids Res 25: 1469–1475 10.1093/nar/25.8.1469 PMC146616 9092651

[9] Sheplock R, Recinos DA, Mackow N, Dietrich LEP, Chander M (2013) Species-specific residues calibrate SoxR sensitivity to redox-active molecules. Mol Microbiol 87: 368–381 10.1111/mmi.12101 23205737 PMC3545107

[10] Greenberg JT, Monach P, Chou JH, Josephy PD, Demple B (1990) Positive control of a global antioxidant defense regulon activated by superoxide-generating agents in *Escherichia coli* . Proc Natl Acad Sci USA 87: 6181–6185 10.1073/pnas.87.16.6181 1696718 PMC54496

[11] Tsaneva IR, Weiss B (1990) *soxR*, a locus governing a superoxide response regulon in *Escherichia coli* K-12. J Bacteriol 172: 4197–4205. 1695893 10.1128/jb.172.8.4197-4205.1990 PMC213242

[12] Amábile-Cuevas CF, Demple B (1991) Molecular characterization of the *soxRS* genes of *Escherichia coli*: two genes control a superoxide stress regulon. Nuc Acids Res 19: 4479–4484 10.1093/nar/19.16.4479 PMC328637 1653416

[13] Wu J, Weiss B (1991) Two divergently transcribed genes, soxR and soxS, control a superoxide response regulon of *Escherichia coli* . J Bacteriol 173: 2864–2871. 1708380 10.1128/jb.173.9.2864-2871.1991 PMC207867

[14] Pomposiello PJ, Bennik MH, Demple B (2001) Genome-wide transcriptional profiling of the *Escherichia coli* responses to superoxide stress and sodium salicylate. J Bacteriol 183: 3890–3902 10.1128/JB.183.13.3890-3902.2001 11395452 PMC95271

[15] Palma M, Zurita J, Ferreras JA, Worgall S, Larone DH, et al (2005) *Pseudomonas aeruginosa* SoxR does not conform to the archetypal paradigm for SoxR-dependent regulation of the bacterial oxidative stress adaptive response. Infect Immun 73: 2958–2966 10.1128/IAI.73.5.2958-2966.2005 15845502 PMC1087365

[16] Park W, Peña-Llopis S, Lee Y, Demple B (2006) Regulation of superoxide stress in *Pseudomonas putida* KT2440 is different from the SoxR paradigm in *Escherichia coli* . Biochem Biophys Res Commun 341: 51–56 10.1016/j.bbrc.2005.12.142 16412384

[17] Dietrich LEP, Price-Whelan A, Petersen A, Whiteley M, Newman DK (2006) The phenazine pyocyanin is a terminal signalling factor in the quorum sensing network of *Pseudomonas aeruginosa* . Mol Microbiol 61: 1308–1321 10.1111/j.1365-2958.2006.05306.x 16879411

[18] Singh AK, Shin J-H, Lee K-L, Imlay JA, Roe J-H (2013) Comparative study of SoxR activation by redox-active compounds. Mol Microbiol 90: 983–996 10.1111/mmi.12410 24112649 PMC3872530

[19] Sciara G, Kendrew SG, Miele AE, Marsh NG, Federici L, et al (2003) The structure of ActVA-Orf6, a novel type of monooxygenase involved in actinorhodin biosynthesis. EMBO J 22: 205–215 10.1093/emboj/cdg031 12514126 PMC140106

[20] Huang J, Lih CJ, Pan KH, Cohen SN (2001) Global analysis of growth phase responsive gene expression and regulation of antibiotic biosynthetic pathways in *Streptomyces coelicolor* using DNA microarrays. Genes Dev 15: 3183–3192 10.1101/gad.943401 11731481 PMC312833

[21] Kieser T, Bibb MJ, Buttner MJ, Chater KF, Hopwood DA (2000) Practical *Streptomyces* genetics. Norwich, England: The John Innes Foundation.

[22] Bentley SD, Chater KF, Cerdeño-Tárraga A-M, Challis GL, Thomson NR, et al (2002) Complete genome sequence of the model actinomycete *Streptomyces coelicolor* A3(2). Nature 417: 141–147 10.1038/417141a 12000953

[23] Li H, Durbin R (2009) Fast and accurate short read alignment with Burrows-Wheeler transform. Bioinformatics 25: 1754–1760 10.1093/bioinformatics/btp324 19451168 PMC2705234

[24] Quinlan AR, Hall IM (2010) BEDTools: a flexible suite of utilities for comparing genomic features. Bioinformatics 26: 841–842 10.1093/bioinformatics/btq033 20110278 PMC2832824

[25] Giardine B, Riemer C, Hardison RC, Burhans R, Elnitski L, et al (2005) Galaxy: a platform for interactive large-scale genome analysis. Genome Res 15: 1451–1455 10.1101/gr.4086505 16169926 PMC1240089

[26] Blankenberg D, Kuster von G, Coraor N, Ananda G, Lazarus R, et al (2010) Galaxy: a web-based genome analysis tool for experimentalists. Curr Protoc Mol Biol Chapter 19: 19.10.1–.10.21 10.1002/0471142727.mb1910s89 PMC4264107 20069535

[27] Goecks J, Nekrutenko A, Taylor J (2010) Galaxy Team (2010) Galaxy: a comprehensive approach for supporting accessible, reproducible, and transparent computational research in the life sciences. Genome Biol 11: R86–R86 10.1186/gb-2010-11-8-r86 20738864 PMC2945788

[28] Anders S, Huber W (2010) Differential expression analysis for sequence count data. Genome Biol 11: R106 10.1186/gb-2010-11-10-r106 20979621 PMC3218662

[29] Altschul SF, Gish W, Miller W, Myers EW, Lipman DJ (1990) Basic local alignment search tool. J Mol Biol 215: 403–410 10.1016/S0022-2836(05)80360-2 2231712

[30] Rozen S, Skaletsky H (2000) Primer3 on the WWW for general users and for biologist programmers. Methods Mol Biol 132: 365–386. 10547847 10.1385/1-59259-192-2:365

[31] Cock PJA, Antao T, Chang JT, Chapman BA, Cox CJ, et al (2009) Biopython: freely available Python tools for computational molecular biology and bioinformatics. Bioinformatics 25: 1422–1423 10.1093/bioinformatics/btp163 19304878 PMC2682512

[32] Floriano B, Bibb M (1996) afsR is a pleiotropic but conditionally required regulatory gene for antibiotic production in *Streptomyces coelicolor* A3(2). Mol Microbiol 21: 385–396 10.1046/j.1365-2958.1996.6491364.x 8858592

[33] Rigali S, Derouaux A, Giannotta F, Dusart J (2002) Subdivision of the helix-turn-helix GntR family of bacterial regulators in the FadR, HutC, MocR, and YtrA subfamilies. J Biol Chem 277: 12507–12515 10.1074/jbc.M110968200 11756427

[34] Robinson JT, Thorvaldsdottir H, Winckler W, Guttman M, Lander ES, et al (2011) Integrative genomics viewer. Nat Biotechnol 29: 24–26 10.1038/nbt.1754 21221095 PMC3346182

[35] Thorvaldsdottir H, Robinson JT, Mesirov JP (2013) Integrative Genomics Viewer (IGV): high-performance genomics data visualization and exploration. Brief Bioinformatics 14: 178–192 10.1093/bib/bbs017 22517427 PMC3603213

