# Supplementary material for: RNA-Seq Analysis Reveals a Six-Gene SoxR Regulon in Streptomyces coelicolor
Source: PLoS One. 2014 Aug 27;9(8):e106181. doi: 10.1371/journal.pone.0106181 (PMC4146615; doi:10.1371/journal.pone.0106181)
Supplement: Figure S1 — Complementation analysis to further assess the SoxR-dependence of genes identified by RNA-Seq. (DOCX) [file pone.0106181.s001.docx]

**Figure S1. Complementation analysis to further assess the SoxR-dependence of genes identified by RNA-Seq.** qRT-PCR was performed on RNA isolated from WT/pSET152, Δ*soxR*/pSET152, and a Δ*soxR* strain complemented with wild-type *soxR* (pSoxR), that were grown on R2YE plates for 3 days. The expression levels of all genes were standardized to the level of the constitutively expressed housekeeping sigma factor, *hrdB,* and normalized to expression in WT/pSET152.
