# Supplementary material for: RNA-Seq Analysis Reveals a Six-Gene SoxR Regulon in Streptomyces coelicolor
Source: PLoS One. 2014 Aug 27;9(8):e106181. doi: 10.1371/journal.pone.0106181 (PMC4146615; doi:10.1371/journal.pone.0106181)
Supplement: Figure S2 — Sequence alignment of SCO0320ext and SCO0320 with the quinone oxidoreductase SLI_0274 from S. lividans. (DOCX) [file pone.0106181.s002.docx]

**A.**

110

0320ext ATGCATGCGATCCACGTGGCCTCGTTCGGTGCTCCGGACGTGCTGAGCCTGGTCGACCTGCCCGATCCCGTGCCCGGCCCCGGCCAGGTCGTCGTCGGTATGGTCGCGGC

SLI_0274 ATGCATGCGATCCACGTGGCCTCGTTCGGTGCTCCGGACGTGCTGACCCTGGTCGACCTGCCCGATCCCGTGCCCGGCCCCGGCCAGGTCGTCGTCGGTATGGTCGCGGC

SCO0320 --------------------------------------------------------------------------------------------------------------

*

220

0320ext CGACGTCATCTTCCTGGACACGCTGCTGCGCGGCGGCTGGGGTCAGGACTTCTTCCCGCGCACGCTGCCGTACGTGCCGGGTGGCGGCGGGGCGGGCGAGGTGCTGGCAG

SLI_0274 CGACGTCATCTTCCTGGACACGCTGCTGCGCGGCGGCTGGGGTCAGGACTTCTTCCCGCGCACGCTGCCGTACGTGCCGGGTGGCGGCGGGGCGGGCGAGGTGCTGGCAG

SCO0320 --------------------------------------------------------------------------------------------------------------

330

0320ext TCGGTGACGGAGTGGACCCGGGCTGGGTCGGCCGGAGTGTGGTCGTGAGAACCGGGACCGGATACGCCGAACAGGTCGTCGCGAGTGCGCAGGAGATCATGCCGGTTCCC

SLI_0274 TCGGTGACGGAGTGGACCCGGGCTGGGTCGGCCGGAGTGTGGTCGTGAGAACCGGGACCGGATACGCCGAACAGGTCGTCGCGAGTGCGCAGGAGATCATGCCGGTTCCC

SCO0320 --------------------------------------------------------------------------------------------------------------

440

0320ext GCTGGGCTGGCCGCGGTGACGGCCGCCGCACTGGTGCACGACTGCGTGACCGCTCTCGGCTTCCACCGGCTGGGGGCGCCACAGAAGGGGGAATGGGTCCTGGTCTCGGC

SLI_0274 GCTGGGCTGGCCGCGGTGACGGCCGCCGCACTGGTGCACGACGGCGTGACCGCTCTCGGCTTCCACCGGCTGGGGGCGCCGCAGAAGGGGGAATGGGTCCTGGTCTCGGC

SCO0320 --------------------------------------------------------------------------------------------------------------

* *

550

0320ext GGCGGCCGGCGGGGCGGGCACCCTGCTGGTGCAGTTGGCGGTCGACGCGGGGG-CCGGGTGGTGGCCGCCGCGTCCAGCGACGCCAAGCTGGCTCTGGCCCGCGATCTGG

SLI_0274 GGCGGCCGGCGGGGCGGGCACCCTGCTGGTGCAGTTGGCGGTCGACGCGGGGGCCCGGGTGGTGGCCGCCGCGTCCAGCGACGCCAAGCTGGCTCTGGCCCGCGATCTGG

SCO0320 ----------------------------------------------------------GTGGTGGCCGCCGCGTCCAGCGACGCCAAGCTGGCTCTGGCCCGCGATCTGG

:

660

0320ext GCGCCGAGGTCGTCGTCGACTACACGCGGGCGGACTGGGTCGAGCGGGTGCGCGAGGCGACCGGTGGCGGCGCCGCGCTCGTCTACGACGGCGCGGGCGGGGCGCTCGGT

SLI_0274 GCGCCGAGGTCGTCGTCGACTACACGCGGGCGGACTGGGTCGAGCGGGTGCGCGAGGCGACCGGTGGCGGCGCCGCGCTCGTCTACGACGGCGCGGGCGGGGCGCTCGGT

SCO0320 GCGCCGAGGTCGTCGTCGACTACACGCGGGCGGACTGGGTCGAGCGGGTGCGCGAGGCGACCGGTGGCGGCGCCGCGCTCGTCTACGACGGCGCGGGCGGGGCGCTCGGT

770

0320ext GCGACCAGCGTCGACGCGCTCGCGGACGGCGGGCGCTTCGTCACCTACGGGACCGCCGACGGATTCGCCGCCCCGGACCGGGAATCCGCCGCGCGCCGGGGCATCCGGCT

SLI_0274 GCGACCAGCGTCGACGCGCTCGCGGACGGCGGGCGCTTCGTCACCTACGGGACCGCCGACGGATTCGCCGCCCCGGACCGGGAATCCGCCGCGCGCCGAGGCATCCGGCT

SCO0320 GCGACCAGCGTCGACGCGCTCGCGGACGGCGGGCGCTTCGTCACCTACGGGACCGCCGACGGATTCGCCGCCCCGGACCGGGAATCCGCCGCGCGCCGGGGCATCCGGCT

*

880

0320ext GCTCATGCCGCTCATGGACGGCCCTCCGGACCAGGAGACCGCCCGGGAACTGCTGGGCCTGGCGCTGGAGAGCGCCGCCGAGGGACGCCTGCGCCCGGCCATCGGCGCCA

SLI_0274 GCTCATGCCGCTCATGGACGGCCCTCCGGACCAGGAGACCGCCCGGGAACTGCTGGGCCTGGCGCTGGAGAGCGCCGCCGAGGGACGCCTGCGCCCGGCCATCGGCGCCA

SCO0320 GCTCATGCCGCTCATGGACGGCCCTCCGGACCAGGAGACCGCCCGGGAACTGCTGGGCCTGGCGCTGGAGAGCGCCGCCGAGGGACGCCTGCGCCCGGCCATCGGCGCCA

957

0320ext CCTACCCGCTGGCGCGGGCCGCGGACGCCCACCGCGCTCTGGCGGCGCGTACGACGGTGGGCAAGTCACTGCTCCTGA

SLI_0274 CCTACCCGCTGGCGCGGGCCGCGGACGCCCACCGCGCTCTGGCGGCGCGTACGACGGTGGGCAAGTCACTGCTCCTGA

SCO0320 CCTACCCGCTGGCGCGGGCCGCGGACGCCCACCGCGCTCTGGCGGCGCGTACGACGGTGGGCAAGTCACTGCTCCTGA

**B.**

***************:********************************************************************************************

SCO0320ext MHAIHVASFGAPDVLSLVDLPDPVPGPGQVVVGMVAADVIFLDTLLRGGWGQDFFPRTLPYVPGGGGAGEVLAVGDGVDPGWVGRSVVVRTGTGYAEQVVASAQEIMP

SLI_0274 MHAIHVASFGAPDVLTLVDLPDPVPGPGQVVVGMVAADVIFLDTLLRGGWGQDFFPRTLPYVPGGGGAGEVLAVGDGVDPGWVGRSVVVRTGTGYAEQVVASAQEIMP

SCO0320 ------------------------------------------------------------------------------------------------------------

****************!****************************************

SCO0320ext VPAGLAAVTAAALVHDCVTALGFHRLGAPQKGEWVLVSAAAGGAGTLLVQLAVDAGAGWWPPRPATPSWLWPAIWAPRSSSTTRGRTGSSGCARRPVAAPRSSTTARA

SLI_0274 VPAGLAAVTAAALVHDGVTALGFHRLGAPQKGEWVLVSAAAGGAGTLLVQLAVDAGARVVAAASSDAKLALARDLGAEVVVDYTRADWVERVREATGGGAALVYDGAG

SCO0320 ----------------------------------------------------------VVAAASSDAKLALARDLGAEVVVDYTRADWVERVREATGGGAALVYDGAG

**************************************************

SCO0320ext GRSVRPASTRSRTAGASSPTGPPTDSPPRTGNPPRAGASGCSCRSWTALRTRRPPGNCWAWRWRAPPRDACARPSAPPTRWRGPRTPTALWRRVRRWASHCS

SLI_0274 GALGATSVDALADGGRFVTYGTADGFAAPDRESAARRGIRLLMPLMDGPPDQETARELLGLALESAAEGRLRPAIGATYPLARAADAHRALAARTTVGKSLLLMGGE

SCO0320 GALGATSVDALADGGRFVTYGTADGFAAPDRESAARRGIRLLMPLMDGPPDQETARELLGLALESAAEGRLRPAIGATYPLARAADAHRALAARTTVGKSLLLMGGE

***********************************************************************************************************

**Figure S2.** Sequence alignment of *SCO0320ext* and *SCO0320* with the quinone oxidoreductase *SLI_0274* from *S. lividans.*

A) Nucleotide alignment of the three genes from *Streptomyces.* Mismatches are indicated by an asterisk below the alignment; a deletion is indicated by a colon. B) Amino acid sequence alignment of the predicted open reading frames of the same genes. Identical residues are indicated by an asterisk; conservative substitutions by a colon; non-conservative substitutions by an exclamation point. *SCO0320ext* and *SLI_0274* are highly conserved in the amino-terminal half (identical amino acids shown above the alignment); while *SCO0320* is identical to the carboxy-terminal half of *SLI_0274* (identical amino acids shown below alignment).
