# Supplementary material for: RNA-Seq Analysis Reveals a Six-Gene SoxR Regulon in Streptomyces coelicolor
Source: PLoS One. 2014 Aug 27;9(8):e106181. doi: 10.1371/journal.pone.0106181 (PMC4146615; doi:10.1371/journal.pone.0106181)
Supplement: Table S1 — Primers used in this study. (DOCX) [file pone.0106181.s003.docx]

**Table S1. Primers used in this study**

| **Promoter probes for EMSA** | **Sequence (5’ → 3’)** | **Promoter** | **Size (bp)** |
| --- | --- | --- | --- |
| 0320p-F | GAACAGGTCGTCGCGAGTG | SCO0320 | 200 |
| 0320p-R | GCGTCGACCGCCAACTGC |  |  |
| 0320extp-F | GACAGTCGTAGGCGGTTCC | SCO0320ext | 200 |
| 0320extp-R | GACCAGGCTCAGCACGTC |  |  |
| 0321p-F | GTACGACGGTGGGCAAGTC | SCO0321 | 171 |
| 0321p-R | CTCTCGCTGCACGCCTGT |  |  |
| 7009p-F | GGACACGGCGGTGTGGCT | SCO7009 | 160 |
| 7009p-R | CCACGCTCGTTCCCGCAC |  |  |
|  | | | |
| **(q)RT-PCR** | **Sequence (5’ → 3’)** | **Amplicon** | **Size (bp)** |
| hrdB-F | CATGCGCTTCGGACTCA | SCO5820  (*hrdB*) | 95 |
| hrdB-R | ACTCGATCTGGCGGATG |  |  |
| 0319-F | AGCGTGTCCAGGAAGATGAC | SCO0319 | 98 |
| 0319-R | GACGTGCTGAGCCTGGTC |  |  |
| 0320ext-F | GTCGGTGACGGAGTGGAC | SCO0320 | 100 |
| 0320ext-R | CATGATCTCCTGCGCACTC |  |  |
| 0321N-F | TGACGAGGACATCCTCACTG | SCO0321 (N-term) | 105 |
| 0321N-R | GACGAGCCGAAGGTGTAGG |  |  |
| 0321C-F | ACCTGCTGTTCGGTGAGTAC | SCO0321 (C-term) | 85 |
| 0321C-R | GTCGAAGCGGGACAGGAAAG |  |  |
| 1177-F | GATACGTCGAGGCCGACA | SCO1177 | 120 |
| 1177-R | TGCTGACGAGGAAGCTGTG |  |  |
| 1178-F | TCAAGGTCCGGCAGGTCTA | SCO1178 (*ecaC*) | 82 |
| 1178-R | CCGTCCTCCTGCTTGGT |  |  |
| 1277-F | CAGCAAGAACGTCCAAGACA | SCO1277 | 83 |
| 1277-R | TCAGGAGGTCGTCGAAGG |  |  |
| 1697-F | TGCCTCAGATTCCAGAGAAGA | SCO1697 (*soxR*) | 105 |
| 1697-R | TCAGACCCTTGGACTCGTAGA |  |  |
| 1734-F | TCTACGACTGGAACGGCATC | SCO1734 | 112 |
| 1734-R | AGGTCCAGACCCTTGAACG |  |  |
| 1909-F | CGCGGTTACCACGTCTACA | SCO1909 (*ecaB*) | 101 |
| 1909-R | CGGTGGGTCTTGACGAAG |  |  |
| 2055-F | CAGGATCCAGCACTTCGAG | SCO2055 | 103 |
| 2055-R | GTCGTCGGTGAAGGTGAAC |  |  |
| 2478-F | GAGATCACCCCGAAACTGG | SCO2478 | 104 |
| 2478-R | AAGTGCCAGTCGATGACGTT |  |  |
| 4020-F | CTCAGAGCGTCCCGACAT | SCO4020 | 112 |
| 4020-R | CTCGTCGTCCACTACAAGCA |  |  |
| 4021-F | ACTACCGCGTCGAGTACCAC | SCO4021 | 82 |
| 4021R | AGGACCAGGGTGCTGATG |  |  |
| 4157-F | ACGTACAAGGCGATCCAGAC | SCO4157 | 94 |
| 4157-R | AGTTGATGCCGATGATGTTG |  |  |
| 4266-F | GATGGGCATCCTCCAGTTC | SCO4266 (*ecaD*) | 104 |
| 4266-R | CGTTCTTCGCGTACTGCAC |  |  |
| 4671-F | GGTGACCGAACTCCAGCA | SCO4671 | 105 |
| 4671-R | AGGAGGGCGCCCAGTAAG |  |  |
| 4680-F | ACTGCCTCAACCTCTTCAGC | SCO4680 | 104 |
| 4680-R | GACGACCGACAGCAGCTC |  |  |
| 4681-F | GGACAAGCTCGCGAAGAC | SCO4681 | 115 |
| 4681-R | CAGAATCTGGCCGTTGACC |  |  |
| 4682-F | CTTTCGCCCACTTCAAGGT | SCO4682 | 85 |
| 4682-R | GCGTAGAGGTCGGTGGTG |  |  |
| 4683-F | ATCTTCAAGAACGCGCTGAC | SCO4683 | 114 |
| 4683-R | CATGAAGGACTGGCAGAACC |  |  |
| 4684-F | AAAAGGGATTCGGCTTCATC | SCO4684 | 120 |
| 4684-R | TGTCGAAGTTCACCTTCTGG |  |  |
| 4685-F | GGACACGTCACCGTCCTAGT | SCO4685 | 117 |
| 4685-R | GCGGTGCAGATAGTCCTTGT |  |  |
| 4687-F | CCACCTGCACTTCCATGTC | SCO4687 | 98 |
| 4687-R | AGGCACTGACGTCATCGAG |  |  |
| 4690-F | CTCTACCCGGCGTACTGGT | SCO4690 | 104 |
| 4690-R | AGGTCGAGCAGGACCTCAC |  |  |
| 4692-F | CTCGTTACACGGAGGCACTG | SCO4692 | 81 |
| 4692-R | GTACGTCGAACTCCCACCAG |  |  |
| 6165-F | GAGGTCCGGGTCAAGCTG | SCO6165 | 103 |
| 6165-R | ACAGATGGCAGTTGCCGTAG |  |  |
| 6502-F | CCGAGCGGATCTACTACGAC | SCO6502 | 119 |
| 6502-R | CAAGAGCTCGTCCTCCTGTC |  |  |
| 7008-F | TCGTCTCCCGTCTCAACAA | SCO7008 (*ecaA*) | 124 |
| 7008-R | CAGGACAGGGTCAGCATCA |  |  |
|  | | | |
| **Sequencing** | **Sequence (5’ → 3’)** |  | **Size (bp)** |
| 0320extseq-F | GTCGGTGACGGAGTGGAC |  | 494 |
| 0320extseq-R | GGTCCCGTAGGTGACGAAG |  |  |
