# Supplementary material for: RNA-Seq Analysis Reveals a Six-Gene SoxR Regulon in Streptomyces coelicolor
Source: PLoS One. 2014 Aug 27;9(8):e106181. doi: 10.1371/journal.pone.0106181 (PMC4146615; doi:10.1371/journal.pone.0106181)
Supplement: Table S2 — Genes identified by RNA-Seq as upregulated in Δ soxR compared to WT in stationary phase. (DOCX) [file pone.0106181.s004.docx]

**Table S2. Genes identified by RNA-Seq as upregulated in Δ*soxR* compared to WT in stationary phase^a^**

| ***SCO* Number** | **Increase in Δ*soxR* versus WT on Day 3^b^** | | **Decrease in WT on Day 3 vs. Day 1^c^** | | **Predicted function^f^** |
| --- | --- | --- | --- | --- | --- |
|  | **Fold change** | **q-value^d^** | **Fold change^e^** | **q-value^d^** |  |
| *4681* | 10 | 3e-17 | 2 | 3e-1 | Dehydrogenase |
| *4684* | 10 | 2e-23 | 3 | 7e-2 | Cold shock protein |
| *4694* | 8 | 2e-1 | <1 | 1e0 | Hypothetical protein |
| *4671* | 8 | 3e-7 | <-1 | 9e-1 | Putative lysR-family regulatory protein |
| *4999* | 8 | 3e-2 | -2 | 7e-1 | Hypothetical protein |
| *4682* | 7 | 1e-7 | 3 | 2e-1 | Tautomerase |
| *4692* | 7 | 6e-14 | <1 | 8e-1 | Hypothetical protein |
| *2055* | 7 | 3e-16 | 2 | 1e-2 | Membrane associated oxidoreductase |
| *4686* | 7 | 4e-14 | <-1 | 4e-1 | Hypothetical protein |
| *4693* | 7 | 1e-9 | 1 | 6e-1 | Membrane protein |
| *1803* | 6 | 2e-1 | 2 | 1e0 | Oxidoreductase |
| *4690* | 6 | 2e-5 | -3 | 4e-3 | Membrane protein |
| *4679* | 6 | 7e-13 | <1 | 6e-1 | Hypothetical protein |
| *1282* | 6 | 8e-3 | 3 | 8e-1 | Oxidoreductase |
| *4685* | 6 | 5e-13 | <-1 | 10e-1 | DEAD-box RNA helicase |
| *4672* | 6 | 10e-11 | -1 | 2e-1 | Secreted protein |
| *4683* | 6 | 2e-13 | <-1 | 8e-1 | GdhA, NADP-specific glutamate dehydrogenase |
| *4687* | 6 | 7e-13 | <-1 | 7e-1 | Hypothetical protein |
| *4689* | 6 | 5e-7 | -2 | 7e-3 | Hypothetical protein |
| *7494* | 6 | 4e-1 | <1 | 1e0 | Membrane protein |
| *3657* | 6 | 7e-7 | <1 | 1e0 | Hypothetical protein |
| *4678* | 6 | 2e-13 | 1 | 4e-1 | DNA-binding protein |
| *4680* | 5 | 3e-12 | <1 | 6e-1 | DNA-binding protein |
| *1804* | 5 | 9e-2 | 1 | 8e-1 | S-adenosylmethionine:tRNA ribosyltransferase-isomerase |
| *7130* | 5 | 5e-1 | -1 | 9e-1 | Hypothetical protein |
| *6508* | 5 | 4e-1 | -1 | 8e-1 | Gas vesicle synthesis protein |
| *6502* | 5 | 9e-2 | -2 | 1e-1 | Gas-vesicle synthesis protein |
| *4691* | 5 | 2e-2 | -1 | 6e-1 | Membrane protein |
| *1283* | 5 | 5e-2 | 3 | 6e-1 | Cyclase/dehydrase |
| *2695* | 4 | 4e-2 | <-1 | 1e0 | Serine/threonine kinase |
| *1277* | 4 | 3e-8 | 6 | 2e-6 | Tape-measure protein |
| *1278* | 4 | 5e-7 | 4 | 2e-3 | ATP/GTP binding protein |
| *6501* | 4 | 1e-2 | -2 | 3e-1 | Gas vesicle synthesis protein |
| *0827* | 4 | 4e-1 | -1 | 7e-1 | Hypothetical protein |

^a^ Genes are organized in decreasing order of SoxR-dependence as determined by RNA-Seq.

^b^  RNA for this comparison was obtained from 3-day old WT or Δ*soxR* cultures, both of which were blue-pigmented.

^c^ RNA for this comparison was obtained from 3-day old WT (blue-pigmented) or 1-day old WT (unpigmented) cultures.

^d^ q-value is the false discovery rate; the probability that the result is a false positive.

^e^ A negative value in this column indicates that gene expression was higher in 3-day old WT culture than in 1-day old WT culture.

^f^ Predicted functions of genes were obtained from StrepDB (http://strepdb.Streptomyces.org.uk).
