# Supplementary material for: RNA-Seq Analysis Reveals a Six-Gene SoxR Regulon in Streptomyces coelicolor
Source: PLoS One. 2014 Aug 27;9(8):e106181. doi: 10.1371/journal.pone.0106181 (PMC4146615; doi:10.1371/journal.pone.0106181)
Supplement: Table S3 — Validation of RNA-Seq data reported in Table S2 by quantitative RT-PCR. (DOCX) [file pone.0106181.s005.docx]

**Table S3. Validation of RNA-Seq data reported in Table S2 by quantitative RT-PCR^a^**

| ***SCO* Number** | **Fold increase in Δ*soxR* versus WT^b^** | **Fold increase in Δ*act* versus WT^c^** | **Predicted function** |
| --- | --- | --- | --- |
| *4681* | 2 | 1 | Dehydrogenase |
| *4684* | 16 | 2 | Cold shock protein |
| *4671* | 2 | 2 | Putative lysR-family regulatory protein |
| *4682* | 4 | 1 | Tautomerase |
| *4692* | 3 | 2 | Hypothetical protein |
| *2055* | 4 | 16 | Membrane associated oxidoreductase |
| *4690* | 2 | 3 | Membrane protein |
| *4685* | 2 | 2 | DEAD-box RNA helicase |
| *4683* | 2 | 2 | GdhA, NADP-specific glutamate dehydrogenase |
| *4687* | 16 | ND | Hypothetical protein |
| *4680* | 1 | 2 | DNA-binding protein |
| *6502* | 5 | 14 | Gas-vesicle synthesis protein |
| *1277* | 4 | 4 | Tape-measure protein |

**^a^** RNA for qRT-PCR validation was obtained from independent biological samples.

^b^ Differential gene expression in WT and Δ*soxR* in 3-day old cultures assessed by qRT-PCR. Gene expression was standardized to the housekeeping sigma factor, *hrdB,* and normalized to WT.

^c^ Differential gene expression in WT and the Δ*act* strain (M511) in 3-day old cultures assessed by qRT-PCR. Gene expression was standardized to the housekeeping sigma factor, *hrdB,* and normalized to WT.
